# HPV DNA in saliva from patients with SCC of the head and neck is specific for p16-positive oropharyngeal tumours

**DOI:** 10.1186/s40463-016-0179-6

**Published:** 2017-01-06

**Authors:** Jason K. Wasserman, Ryan Rourke, Bibianna Purgina, Lisa Caulley, Jim Dimitroulakis, Martin Corsten, Stephanie Johnson-Obaseki

**Affiliations:** 1 Department of Pathology and Laboratory Medicine, Division of Anatomical Pathology, The University of Ottawa and The Ottawa Hospital, Ottawa, Ontario Canada; 2 Department of Otolaryngology - Head and Neck Surgery, The University of Ottawa and The Ottawa Hospital, Ottawa Hospital-General Campus S3, 501 Smyth Rd, Ottawa, Ontario K1H 8L6 Canada; 3 Ottawa Hospital Research Institute, Ottawa, Ontario Canada; 4 Aurora St. Luke’s Hospital, Milwaukee, WI USA

**Keywords:** Human papillomavirus, Oropharyngeal cancer, Head and neck cancer, Screening

## Abstract

**Background:**

Human papillomavirus (HPV) is an important cause of head and neck squamous cell carcinoma (HNSCC), especially in young people. These tumours overexpress p16 and respond well to treatment. The rapid detection of HPV in patients with HNSCC may expedite treatment when p16 status is not immediately available.

**Methods:**

Saliva-based DNA collection kits and nested polymerase chain reaction (PCR) were used to determine the HPV status of 62 individuals with biopsy-proven HNSCC. Immunohistochemistry was used to determine tumour p16 status.

**Results:**

A total of 62 patients were included in the study. Twenty-nine samples (47%) were positive for HPV DNA, the majority of which were high risk (HR) subtypes (79%). Patients who tested positive for HR HPV were more likely to have a tumour arising in the oropharynx compared to a non-oropharyngeal site (74 vs 26%; *p* = 0.003). A positive HR HPV saliva assay was 100% specific (95% CI 59–100%) and had a 100% positive predictive value (95% CI 75–100%) for a p16 positive tumour arising in the oropharynx. In contrast, a negative HR HPV assay had a 96% negative predictive value (95% CI 80–100%) for tumours arising in a non-oropharyngeal site. Independent of site, the saliva assay had a sensitivity of 77% (95% CI 54–91%) and a specificity of 94% (95% CI 77–99%), respectively, for a p16 positive tumour.

**Conclusion:**

We show that a saliva based assay is an effective method for detecting HPV in patients with HNSCC and that a positive HR HPV test is highly specific for p16 positive tumours arising in the oropharynx. This simple and rapid test could be used in cases where a biopsy of the primary tumour is not readily available.

## Background

Human papilloma virus (HPV) is a small, non-enveloped DNA virus capable of infecting keratinocytes in the skin or mucous membranes [1]. It is a ubiquitous virus with some estimates suggesting that 80% of sexually active women will be exposed to the virus during their lifetime. In the vast majority of cases, the infection is cleared by the immune system. However, exposure to a high-risk HPV (HR HPV) subtype can lead to persistent infection which, if left untreated, can progress to cancer [2]. The link between high HR HPV and cancer is well documented in the cervix, vulva, vagina, penis, and anal canal and recent studies have also shown that HR HPV is also a risk factor for oropharyngeal squamous cell carcinoma (HNSCC) [3]. Indeed, while HNSCC associated with traditional risk factors such as smoking and alcohol have been steadily on the decline for decades, cancers associated with HR HPV infection are on the rise, especially among young people in industrialized countries [4, 5].

HPV infects the basal layer of the epithelium where it can remain dormant for a variable period of time before entering a productive phase. Clearance is T-cell mediated and individuals with impaired cellular immunity have a higher risk of progression to cancer [1]. When expressed, the HPV oncogenes E6 and E7 induce the degradation of the tumour suppressors p53 and pRB, which leads to uncontrolled cellular proliferation [6, 7]. Because pRB normally inhibits the expression of p16, cells lacking pRB over-express p16 which can be detected by immunohistochemistry and is commonly used as a surrogate marker for HR HPV infection. HPV DNA can be detected in saliva samples when cells are actively producing viral particles or when infected cells die and release the virus back into the saliva [8].

Patients with tumours over-expressing p16 respond well to treatment and the rapid detection of HPV, especially a high risk subtype, in a patient with HNSCC may expedite treatment [9]. Furthermore, the combination of HR HPV DNA and p16 immunoreactivity may be a better predictor of outcome than either marker alone [10]. With those benefits in mind, we employed a novel saliva based DNA assay in order to test the hypothesis that the presence of HR HPV DNA predicts p16 positivity in patients with biopsy-proven SCC of the head and neck.

## Methods

### Study population

This study was approved by the Ottawa Hospital Research Ethics board. All patients who were seen at the Ottawa Regional Cancer Centre (ORCC) with biopsy proven squamous cell carcinoma of the upper aerodigestive tract (excluding esophageal cancer) were approached to participate in the study. Exclusion criteria included patients less than 18 years of age and those not able to consent (due to dementia or other reasons) or those unable to expectorate (poor performance status or very advanced age).

### Study design

Saliva samples were collected from consenting patients using the Oragene®•DNA kit (OG-500) manufactured by DNA Genotek, Inc. (www.dnagenotek.com). Saliva was collected as per the manufacturer’s recommendation. Each sample was coded with a study number and sent for analysis at the Ottawa Hospital Research Institute. The quality and quantity of extracted DNA was determined by β-globin specific PCR as described previously [11]. Screening for the presence of HPV DNA was done using a nested PCR approach consisting of the PGMY09/11 primer set (primary PCR) and the GP5+/6+ primer set (secondary PCR) as described previously [12, 13]. Briefly, 5 μL of each sample was amplified with PGMY09/11 primers (5 pmol each) and amplifications were performed in a GeneAmp PCR System 9700 (Applied Biosystems, Carlsbad, CA) using the following specifications: 95 °C for 2 min, 40 cycles of 95 °C for 1 min, 55 °C for 1 min, and 72 °C for 1 min. This was followed by a final extension of 10 min at 72 °C, and then storage at 4 °C. The secondary PCR consisted of amplification of 2 μL of the primary PCR product using the GP5+/6+ primers (5 pmol each). Amplifications were performed in the same machine as described above using the following specifications: 94 °C for 2 min, and 40 cycles of 94 °C for 45 s, 48 °C for 4 s, 38 °C for 30 s, 42 °C for 5 s, 66 °C for 5 s, and 71 °C for 1.5 min. This was followed by a final extension of 10 min at 72 °C, and then storage at 4 °C. The PCR products were electrophoresed using a 2% low-melting point gel (NuSieve GTG Agarose), stained with ethidium bromide and photographed under UV-transillumination. The bands from any samples that were PCR positive were isolated and purified using the Qiaquick Gel Extraction Kit (Qiagen, Toronto, ON) following manufacturers protocol. The purified PCR products were then submitted for automated DNA sequencing for forward and reverse sequencing (StemCore Sequencing Facility, Ottawa, ON). The sequences were compared with HPV genomes deposited in the NCBI-GenBank using the BLAST program (NCBI).

### Immunohistochemistry

Immunohistochemical analysis for p16 (dilution 1:500, clone SC-56330, Leica) was performed on formalin fixed tissue sections using the BOND-MAX automated system (Leica Microsystems, Wetzlar, German). Tissue sections were subjected to epitope retrieval using a Bond epitope retrieval solution appropriate for the primary antibody.

### Statistical analysis

Statistical analysis was performed using SPSS 21 for Windows (IBM, USA). For the purpose of this analysis, the HPV subtypes were divided into low- and high-risk categories according to the recent classification of the International Agency for Research on Cancer. Specifically, the following HPV subtypes were defined as high-risk based on their known carcinogenic potential in humans: types 16, 18, 31, 33, 35, 39, 45, 51, 52, 56, 58, and 59; all others were defined as low-risk [2]. Similarly, the primary tumour subsite was divided into oropharynx (base of tongue and tonsil) and non-oropharynx (all other sites). A Chi square or Fisher’s exact test was used to compare categorical variables. The sensitivity and specificity of the HPV test for predicting a p16 positive tumour was calculated with a 95% confidence interval. A test was considered statistically significant when the *p*-value was <0.05.

## Results

Out of the 70 patients invited to participate in the study, 62 gave consent. All participants who gave consent to participate were included in the final analysis. The clinical and pathological characteristics of the patients included in this cohort are shown in Table 1. The majority of the patients in this study were male (74%) and the mean age at time of first pathological diagnosis was 58 years (range 32–83 years). Oral cavity and tonsil were the most common tumour sites (32% of cases each). Three patients presented with metastatic disease from an unknown primary and one patient was diagnosed with carcinoma in situ. The tumour stage varied greatly between patients but patients with an oropharynx tumour (base of tongue or tonsil) were much more likely to present with node positive disease compared to other sites (67 vs 22%; *p* = 0.001).

**Table 1:** Clinical and pathological characteristics of patients with squamous cell carcinoma of the head and neck

Sex	*N* (%)
Male	46 (74)
Female	16 (26)
Tumour location	
Oral cavity	20 (32)
Base of tongue	7 (11)
Tonsil	20 (32)
Larynx	11 (18)
Nasopharynx	1 (2)
Unknown	3 (5)
Positive for HPV by saliva test	29 (47)
Low-risk HPV	6 (21)
High-risk HPV	23 (79)
T-stage	
X	4 (6)
is	1 (2)
T1	17 (27)
T2	24 (38)
T3	12 (19)
T4	4 (7)
Lymph node positive	27 (44)
Metastatic disease	4 (7)
p16 positive by immunohistochemistry^a^	22 (36)

^a^p16 status was only available for 53 patients

Immunohistochemistry for p16 was performed on 53 tumour samples; material was not available for immunohistochemical analysis in the other 9 cases. Of the 53 tumour samples tested, 22 (36%) were positive for p16 overexpression. All p16 positive tumours demonstrated strong and diffuse nuclear and cytoplasmic staining. The majority of tumours arising in the oropharynx were positive for p16 (71%) while only 5 tumours (17%) arising in a non-oropharyngeal site were positive for p16. Non-oropharyngeal and p16 positive tumours were located in the lateral tongue, floor of mouth, soft palate, larynx, and nasopharynx.

Of the 62 saliva samples tested, 29 (47%) were positive for HPV DNA, the vast majority of which were a high-risk type (79%). The specific subtypes isolated are shown in Table 2. The most common subtype isolated was HPV-16 which was present in 29% of all patients tested and 62% of saliva HPV positive patients. All other subtypes occurred in a small minority of patients.

**Table 2:** HPV subtypes detected by saliva based DNA assay

HPV subtype	Frequency	Percentage of all patients	Percentage of patients positive for HPV
6	1	1.6	3.4
16	18	29	62.1
27	1	1.6	3.4
33	2	3.2	6.9
56	1	1.6	3.4
58	1	1.6	3.4
59	1	1.6	3.4
66	1	1.6	3.4
72	1	1.6	3.4
73	1	1.6	3.4
76	1	1.6	3.4

The clinical and pathological factors associated with a positive HPV saliva test are shown in Table 3. Compared to females, a higher proportion of males were observed to have HPV DNA present in their saliva, although the difference did not reach statistical significance (54 vs 25%; *p* = 0.08). Patients who tested positive for HPV were more likely to present with node positive disease (63 vs 34%; *p* = 0.010) as were patients with p16 positive tumours (82 vs 13%; *p* < 0.001).

**Table 3:** Clinical and pathological factors associated with HPV status

	HPV status		
	Negative	Positive	*p*-value
Sex			0.08
Male	21	25	
Female	12	4	
Anatomical subsite			0.07
Oropharynx	12	18	
Non-oropharynx	21	11	
Tumour stage			0.18
X	2	3	
T1	7	10	
T2	15	9	
T3	5	7	
T4	4	0	
Lymph node status			0.04
Negative	23	12	
Positive	10	17	
Metastatic disease			1.000
Negative	31	27	
Positive	2	2	
p16 status^a^			<0.001
Negative	27	4	
Positive	4	18	

^a^p16 status was only available for 53 patients

The relationship between HR HPV and p16 status is shown in Table 4. Patients who tested positive for HR HPV were significantly more likely to have a tumour arising in the oropharynx compared to a non-oropharyngeal site (74 vs 26%; *p* = 0.003). Of the 19 patients that tested positive for HR HPV, 2 had tumours that were negative for p16 and both of these tumours were located outside of the oropharynx. Two of the five p16-positive tumours arising outside of the oropharynx were also positive for HR HPV. In our patient population a positive HR HPV saliva assay was 100% specific (95% CI 59–100%) and had a 100% positive predictive value (95% CI 75–100%) for a p16 positive tumour arising in the oropharynx. In contrast, a negative HR HPV assay had a 96% negative predictive value (95% CI 80–100%) for tumours arising in a non-oropharyngeal site. Independent of site, the saliva assay had a sensitivity of 77% (95% CI 54–91%) and a specificity of 94% (95% CI 77–99%), respectively, for a p16 positive tumour.

**Table 4:** Relationship between HR HPV subtype and p16 over-expression

		p16 status						
	HR HPV status	Positive	Negative	*p*-value	Sen (95% CI)	Spe (95% CI)	PPV (95% CI)	NPV (95% CI)
Oropharynx	Positive	13	0	0.001	76 (50–93)	100 (59–100)	100 (75–100)	64 (30–89)
	Negative	4	7					
Non-oropharynx	Positive	5	2	0.002	80 (28–99)	93 (75–99)	67 (22–96)	96 (80–100)
	Negative	1	25					

*Abbreviations*: *HR HPV* High right human papillomavirus, *Sen* Sensitivity, *Spe* Specificity, *PPV* Positive predictive value, *NPV* Negative predictive value

## Discussion

Squamous cell carcinomas arising from head and neck sites are divided into p16-positive and p16-negative tumours in order to guide management and predict response to treatment [9]. In this study we demonstrated used a saliva based assay for detecting HPV DNA in patients with known HNSCC and found that this method correlated with clinical and pathological features associated with p16 positive tumours. In addition, our collection method was extremely easy to use, well accepted by patients and as the kits are stable at room temperature for long periods of time, allowed for batching of samples for future PCR testing.

The prevalence of HPV infection in our study population was 47%, which is considerably higher than a recent meta-analysis in which a prevalence of 21.95% was documented among patients with HNSCC [14]. In this study, however, HPV was only detected in tumour biopsies and it is possible that our assay is more sensitive at detecting low levels of HPV DNA compared to more traditional tissue based methods which also utilize PCR. For example, a saliva based assay could detect HPV DNA present anywhere in the oral cavity or oropharynx. In contrast, a tissue based method would only detect HPV DNA if the virus was present in the tumour cells sampled and the prevalence would be lower if a significant proportion of the tumours arose in the oral cavity.

The prevalence of HPV in our study was also higher in men and in patients with tumours arising from the oropharynx. This is consistent with previous studies in the literature and re-iterates a different epidemiology of HPV-related HNSCC patients compared with traditional HNSCC usually arising in patients with a heavy smoking and alcohol history [15–17]. Interestingly, in our study, 5 tumours arising from a non-oropharygeal sites were positive for p16 over-expression and in 2 of these cases the patients tested positive for salivary HR HPV. This suggests that HPV may play an oncogenic role at least in a subset of non-oropharyngeal HNSCC. Alternatively, HR HPV may not contribute to the pathogenesis of the tumour and the presence of the virus may be a result of the high prevalence of HPV in our population and the multiple risk factors shared among patients with oral cavity and oropharynx SCC.

In our study, patients who tested positive for HR HPV were more likely to present with lymph node positive disease, a finding that is consistent with previous reports that have shown that patients with p16 positive tumours are more likely to present at an advanced stage compared to patients with p16 negative tumours [18]. Paradoxically, patients with p16 positive tumours have overall better prognosis independent of stage [19].

Our assay detected several high-risk types of HPV including 16, 33, 56, 58, and 59. Consistent with previous reports, HPV 16 was the most common subtype. However, in contrast with HPV 18 being the second most common subtype in the literature, in our sample HPV 33 was the only other subtype to be present in more than a single case. This of course may be due to small sample size. The relationship between HPV 16 and HNSCC varies between studies with some suggesting that almost all HPV associated oropharyngeal SCC are positive for HPV 16 [14]. Interestingly, p16 over-expression was detected in all tumours arising from the oropharynx when the patient was also positive for HPV 16. There are two possible explanations for this finding. The first is that exposure to HPV 16 results in a very high rate of persistent infection. This would be consistent with recent findings that HR HPV subtypes tend to be more likely to persist than LR HPV subtypes [20, 21]. An alternative explanation is that infection with HPV 16 results in a high rate of cellular turnover and a high concentration of viral DNA in saliva from affected patients.

Patients with tumours arising in the head and neck frequently present with cervical lymphadenopathy and no evidence of a primary tumour. In our study, HR HPV was strongly associated with tumours arising in the oropharynx. Specifically, a positive HR HPV assay was 100% specific and had a 100% positive predictive value for p16 positive oropharyngeal tumours. In contrast, a positive HR HPV assay had a 96% negative predictive value for tumours arising from a non-oropharyngeal site. Such a result would be particularly useful in the situation where a primary tumour is not identified or an involved lymph node is inaccessible. In this case, a positive HR HPV assay could guide the surgeon to explore the oropharynx and could potentially act as a surrogate marker in the event that a primary tumour is not found and other tissue is unavailable. Also, in situation where confirmation of a base of tongue tumor is required, use of this test could replace the need for a general anaesthetic for tissue confirmation. Finally, several recent studies have shown that a saliva-based assay can be used as a post-treatment surveillance tool because the presence of HPV DNA after treatment is associated with a higher risk of recurrence [22–24]. While the sensitivity of our assay was similar to these studies, it was significantly lower than studies which measured HPV DNA directly in tumoral tissue [25]. As a result of its modest sensitivity but high specificity, a saliva based assay is best suited for confirming the presence of a clinically occult oropharyngeal tumour and monitoring for recurrence of an HPV positive tumour after treatment.

Our study has several limitations. Firstly, we only assessed patients with biopsy-proven SCC and thus the prevalence of HPV in this population is not generalizable to the general population. However, the purpose of this study was to determine if the saliva based assay would correlate with p16 status and to that end we required a population with an established link to p16. Secondly, we did not directly test the tumours for HPV, instead we relied on p16 over-expression as a surrogate marker. The over-expression of p16 in greater than 70% of cells (the method used here in this study) has been shown however to have a 93% sensitivity for HPV infection which would mean we would miss no more than five HPV positive cases in a study of our size [26]. Thirdly, three patients in our study presented with a carcinoma of unknown primary and despite rigorous investigations, the primary site was not identified. We ultimately decided to include these patients in the study as these cases are widely believed to arise from oropharyngeal lesions that disseminate early in their progression without forming a grossly identifiable primary lesion. And finally, p16 status was only available for 53 of the 62 cases presented in our report. The patients in whom we could not determine p16 status were included in the description of HPV prevalence but excluded from the analysis between HPV status and p16 over-expression. While the unavailability of this data decreased the statistical power of the study, it is unlikely that any of our most salient conclusions would have been affected by the addition a small number of additional cases [26].

## Conclusion

Our study shows that a saliva based assay is an effective method for detecting HPV in patients with HNSCC and that a positive HR HPV test was highly specific for p16 positive tumours arising in the oropharynx.

## Abbreviations

HNSCC: Head and neck squamous cell carcinoma
HPV: Human papillomavirus
PCR: Polymerase chain reaction
