# Correction to: HPV DNA in saliva from patients with SCC of the head and neck is specific for p16-positive oropharyngeal tumours

**DOI:** 10.1186/s40463-018-0294-7

**Published:** 2018-08-02

**Authors:** Jason K. Wasserman, Ryan Rourke, Bibianna Purgina, Lisa Caulley, Jim Dimitroulakos, Martin Corsten, Stephanie Johnson-Obaseki

**Affiliations:** 1 Department of Pathology and Laboratory Medicine, Division of Anatomical Pathology, The University of Ottawa and The Ottawa Hospital, Ottawa, ON Canada; 2 Department of Otolaryngology - Head and Neck Surgery, The University of Ottawa and The Ottawa Hospital, Ottawa Hospital-General Campus S3, 501 Smyth Rd, Ottawa, ON K1H 8L6 Canada; 3 Ottawa Hospital Research Institute, Ottawa, ON Canada; 4 Aurora St. Luke’s Hospital, Milwaukee, WI USA

## Correction

Following publication of the original article [1], the authors reported an error in one of the author names. In this Correction the incorrect and correct author names are listed.

Incorrect author name upon publication:

- Jim Dimitroulakis

The correct author name:

- Jim Dimitroulakos
